# Role of SARS-CoV-2 in Altering the RNA-Binding Protein and miRNA-Directed Post-Transcriptional Regulatory Networks in Humans

**DOI:** 10.3390/ijms21197090

**Published:** 2020-09-25

**Authors:** Rajneesh Srivastava, Swapna Vidhur Daulatabad, Mansi Srivastava, Sarath Chandra Janga

**Affiliations:** 1 Department of Biohealth Informatics, School of Informatics and Computing, Indiana University Purdue University, 719 Indiana Ave Ste 319, Walker Plaza Building, Indianapolis, IN 46202, USA; rsrivast@iupui.edu (R.S.); swapdaul@iupui.edu (S.V.D.); 2 Center for Computational Biology and Bioinformatics, Indiana University School of Medicine, 5021 Health Information and Translational Sciences (HITS), 410 West 10th Street, Indianapolis, IN 46202, USA; 3 Department of Medical and Molecular Genetics, Indiana University School of Medicine, Medical Research and Library Building, 975 West Walnut Street, Indianapolis, IN 46202, USA

**Keywords:** SARS-CoV-2, COVID-19, post-transcriptional regulation, RBPs, miRs, RNA

## Abstract

The outbreak of a novel coronavirus SARS-CoV-2 responsible for the COVID-19 pandemic has caused a worldwide public health emergency. Due to the constantly evolving nature of the coronaviruses, SARS-CoV-2-mediated alterations on post-transcriptional gene regulations across human tissues remain elusive. In this study, we analyzed publicly available genomic datasets to systematically dissect the crosstalk and dysregulation of the human post-transcriptional regulatory networks governed by RNA-binding proteins (RBPs) and micro-RNAs (miRs) due to SARS-CoV-2 infection. We uncovered that 13 out of 29 SARS-CoV-2-encoded proteins directly interacted with 51 human RBPs, of which the majority of them were abundantly expressed in gonadal tissues and immune cells. We further performed a functional analysis of differentially expressed genes in mock-treated versus SARS-CoV-2-infected lung cells that revealed enrichment for the immune response, cytokine-mediated signaling, and metabolism-associated genes. This study also characterized the alternative splicing events in SARS-CoV-2-infected cells compared to the control, demonstrating that skipped exons and mutually exclusive exons were the most abundant events that potentially contributed to differential outcomes in response to the viral infection. A motif enrichment analysis on the RNA genomic sequence of SARS-CoV-2 clearly revealed the enrichment for RBPs such as SRSFs, PCBPs, ELAVs, and HNRNPs, suggesting the sponging of RBPs by the SARS-CoV-2 genome. A similar analysis to study the interactions of miRs with SARS-CoV-2 revealed functionally important miRs that were highly expressed in immune cells, suggesting that these interactions may contribute to the progression of the viral infection and modulate the host immune response across other human tissues. Given the need to understand the interactions of SARS-CoV-2 with key post-transcriptional regulators in the human genome, this study provided a systematic computational analysis to dissect the role of dysregulated post-transcriptional regulatory networks controlled by RBPs and miRs across tissue types during a SARS-CoV-2 infection.

## 1. Introduction

An outbreak of coronavirus disease (COVID-19) caused by the newly discovered severe acute respiratory syndrome coronavirus (SARS-CoV-2) started in December 2019 in the city of Wuhan, Hubei Province, China. As of September 16, 2020, COVID-19 has expanded globally, with more than 30 million confirmed cases with over 944,000 deaths worldwide, imposing an unprecedented threat to the public health (https://www.worldometers.info/coronavirus/). In the past two decades, coronavirus outbreaks have resulted in viral epidemics, including a severe acute respiratory syndrome (SARS-CoV) in 2002 with a fatality of 10% and the Middle East respiratory syndrome (MERS-CoV) in 2012 with fatality of 36% [1,2,3,4]. Both SARS-CoV and MERS-CoV were zoonotic viruses originating in bats and camels, respectively [5,6]. However, the recurring emergence of highly pathogenic SARS-CoV, MERS-CoV, and now SARS-CoV-2 have indicated the potential for cross-species transmission of these viruses, thus raising a serious public health concern [7,8]. SARS CoV-2 shares a sequence similarity of 80% and 50% with previously identified SARS-CoV and MERS-CoV, respectively [9,10,11,12]. Since its emergence, rapid efforts have illustrated the molecular features of SARS-CoV-2 that enable it to hijack the host cellular machinery and facilitates its genomic replication and assembly into new virions during the infection process [13,14,15,16].

Coronavirus carries the largest genome among all RNA viruses, ranging from 26 to 32 kilobases in length [12]. This virus has a characteristic “crown”-like appearance under two-dimensional transmission electron microscopy. SARS-CoV-2 is an enveloped positive-sense, single-stranded ribonucleic acid (RNA) coronavirus that belongs to the genus beta-coronavirus. Upon entry in the cell, SARS-CoV-2 RNA is translated into nonstructural proteins (nsps) from two open reading frames (ORFs): *ORF1a* and *ORF1b* [17,18]. The *ORF1a* produces polypeptide 1a, which is cleaved further into 11 nsps, while *ORF1b* yields polypeptide 1ab, which is cleaved into 16 nsps [17,18]. Since SARS-CoV-2 utilizes human machinery to translate its RNA after entry into the cell, it could possibly impact several RNA-binding proteins from the host to bind the viral genome, resulting in altered post-transcriptional regulation. Next, the viral genome is used as the template for replication and transcription, mediated by nonstructural protein RNA-dependent RNA polymerase (RdRP) [18,19]. SARS-CoV-2 encodes four main structural proteins: spike (S), envelope (E), membrane (M), and nucleocapsid (N), which are conserved, and several other accessory proteins (3a, 6, 7a, 7b, 8, and 10), according to the current annotation (GenBank: NC_045512.2) [17,20]. The spike protein, which has evolved the most during the COVID-19 outbreak, enables the virus to bind to angiotensin-converting enzyme 2 (ACE2) on the host cell membrane, following which, it undergoes structural changes and, subsequently, allows the viral genome to make its way inside the host cell [21]. Infections caused by these viruses result in severe pneumonia, fever, and breathing difficulty [22].

A protein-protein interaction map between SARS-CoV-2 and the human proteins published recently has revealed several important targets for drug repurposing [23]. Given the evolving nature of coronaviruses that results in frequent genetic diversity in their genome, it is crucial to identify the regulators in humans that interact with the viral genome and their crosstalk that results in altered regulatory mechanisms in the host during the infection process. Therefore, it is imperative to investigate the interacting post-transcriptional regulators that asset these viral proteins in different tissues.

RNA-binding proteins (RBPs) are a class of proteins in humans that bind to single- or double-stranded RNA and facilitate the formation of ribonucleoprotein complexes [24,25,26]. In addition to RBPs, micro-RNAs (miRs) that belong to a class of noncoding RNAs also interact with target RNAs to regulate the cognate RNA expression [27,28]. Both RBPs and miRs have been widely recognized in regulating the post-transcriptional gene regulatory network in humans [29,30,31]. Dysregulated RBPs and miRs have been shown to contribute significantly to the altered regulatory network in a plethora of diseases, such as cancer, genetic diseases, and viral infections [32,33,34,35,36,37,38]. Previous studies have shown that human RBPs, including the heterogeneous Nuclear Ribonucleoprotein family (hnRNPA1 and hnRNPAQ), polypyrimidine tract-binding protein (PTB), Serine/Arginine-Rich Splicing Factor 7 (SRSF7), and Transformer 2 Alpha Homolog (TRA2A), interact with coronavirus RNA [39,40,41,42,43,44]. Likewise, other reports have demonstrated the potential interaction between human miRNA and the viral genome, including a variety of coronaviruses [41,45,46]. However, the potential RBPs and miRs that interact with SARS-CoV-2 and their implications in viral pathogenesis has been poorly understood.

Currently, there are no proven antiviral therapeutics that are effective against the novel coronavirus. Although the analysis of therapeutic targets for SARS-CoV-2 has been conducted to identify potential drugs by computational methods [47], the targets have not been clinically approved for therapeutic applications. Alternative therapeutics like angiotensin receptor blockers have been identified as tentative target candidates but have shown concerns associated with the loss of angiotensin functions crucial for cells [48]. Therefore, to devise effective therapeutics, there is a need to determine the cellular targets in humans that interact with the virus and result in altered functional outcomes. In this study, we uncovered that several human RBPs and miRNAs harbor abundant binding sites across the SARS-CoV-2 genome, illustrating the titration of post-transcriptional regulators. Interestingly, we show that most of these regulators were predominantly expressed in gonadal tissues, adrenal, pancreas, and blood cells. Overall, this study will bridge the gap in our understanding of the impact of SARS-CoV-2 infection on post-transcriptional regulatory networks.

## 2. Results and Discussion

### 2.1. Protein-Protein Interaction Network Analysis for SARS-CoV-2 Viral Proteins Reveals an Extensive and Direct Set of Interactions with Functionally Important Human RBPs

**Figure 1:**
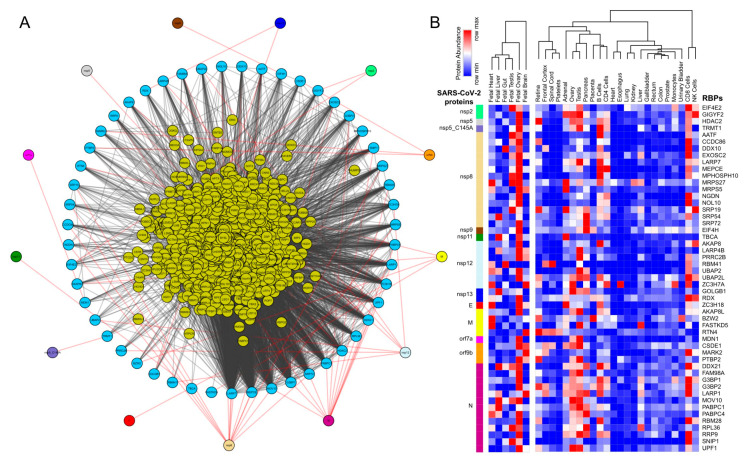
Protein-protein interaction network analysis suggests a direct interaction of human RNA-binding proteins (RBPs) with SARS-CoV-2 viral proteins (**A**) An integrated SARS-CoV-2—human RBP interaction network. We obtained the mass spectrometry (MS)-based SARS-CoV-2 viral protein to the human protein interaction network established in HEK293 cells and integrated with first-neighbor-interacting RBPs (obtained from BioGRID—https://thebiogrid.org). (**B**) Protein abundance of SARS-CoV-2-interacting RBPs across human tissues. Expression data was obtained from the human protein map and row normalized. SARS-CoV-2 proteins were color-coded and highlighted in the network.

We obtained the affinity purification-mass spectrometry (AP-MS)-based SARS-CoV-2 and human proteins interaction network established in HEK293 cells [23] and investigated the human RBPs that directly interact with the viral proteins. Our analysis revealed that SARS-CoV-2-encoded proteins interact directly with 51 human RBPs (Figure 1A). We observed that these primary interacting RBPs were proven to serve several vital functions in the cells, such as polyadenylate binding protein 4 (PABP-4) and Dead-box RNA helicases (DDX21 and DDX10), enzymes involved in translation machinery such as the eukaryotic translation initiation factor 4H (EIF4H), and ribosomal protein L36 (RPL36) (Figure 1A). Among the direct interactors, the highly abundant cytoplasmic PABPs, known to bind the 3′ polyA tail on eukaryotic mRNAs, has previously been reported to interact with polyA tails in bovine coronavirus and the mouse hepatitis virus [49,50,51]. Since SARS-CoV-2 is also composed of polyadenylated RNA, it is likely that the host PABP could modulate the translation of the coronavirus genome through polyA binding. DDX10, another primary interactor observed in the analyzed dataset, has been reported to interact with SARS-CoV-2 nonstructural protein 8 (nsp8) [52], suggesting that the identified host RBPs could be implicated in the regulatory processes of SARS-CoV-2 genome synthesis. EIF4H, also found as one of the primary interactors, was reported to interact with SARS-CoV-2 nonstructural protein 9 (nsp9) in a recently published study [23]. Furthermore, among the immediate interactions, we also found human RBPs such as signal recognition particle 19 (SRP19 and SRP54) and Golgin subfamily B member 1 (GOLGB1) that have been well-recognized for co-translational protein targeting to the membrane and endoplasmic reticulum to Golgi vesicle-mediated transport [53,54] (Figure 1A). These results suggest that several human RBPs that come into direct contact with SARS-CoV-2 proteins could contribute to virus assembly and export and could therefore be implicated as therapeutic targets. However, such findings require in-depth experimental validation in a tissue-specific context to support the functional involvement of the identified RBPs in response to SARS-CoV-2 infection.

Further, we observed that, among the SARS-CoV-2-encoded proteins, the majority of the direct interactions occurred with a range of nonstructural proteins (nsp 2, 5, 8, 9, 11, and 13) that contribute to viral replication and transcription, along with the structural proteins (E, N, and M) (Figure 1A). Therefore, it is likely that the identified human RBPs that interact with the viral proteins assist in the viral pathogenesis. Furthermore, we also observed that ~65% of annotated human RBPs [55] were in immediate neighborhood (obtained from BioGRID [56], shown at the center in Figure 1A) of the virus protein-interacting RBPs and could indirectly regulate the SARS-CoV-2 proteins. Overall, the comprehensive direct and indirect interactions between human RBPs and SARS-CoV-2 proteins are likely to rewire the post-transcriptional gene regulatory mechanisms in human cells.

Next, we examined the abundance of SARS-CoV-2-interacting RBPs across human tissues using the protein expression data from the human proteome map [57]. Our results suggest that a majority of the human RBPs that have direct interactions with SARS-CoV-2 proteins were predominantly expressed in gonadal tissues (testis and ovary) (Figure 1B). These findings agreed with a recent study showing that male reproductive systems are vulnerable to SARS-CoV-2 infection, which was evident by dramatic changes in the sex hormones of the infected patients, suggesting gonadal function impairment [58,59]. Additionally, we also found that these SARS-CoV-2-interacting human RBPs showed relatively higher expressions in immune cell types such as T cells (CD4+ and CD8+) and natural killer (NK) cells that are a part of the innate antiviral immune response (Figure 1B). Our observations are supported by recently published studies suggesting that T cells CD4+ T cells and CD8+ T cells play a significant antiviral role during SARS-CoV-2 infection [20,60,61,62,63]. Overall, the results from this analysis provide a systematic dissection of the potential RBPs in humans interacting with SARS-CoV-2 proteins across tissues.

### 2.2. SARS-CoV-2-Infected Lung Epithelial Cells Exhibit Gene Expression Alterations in Several Immunological and Metabolic Pathways

**Figure 2:**
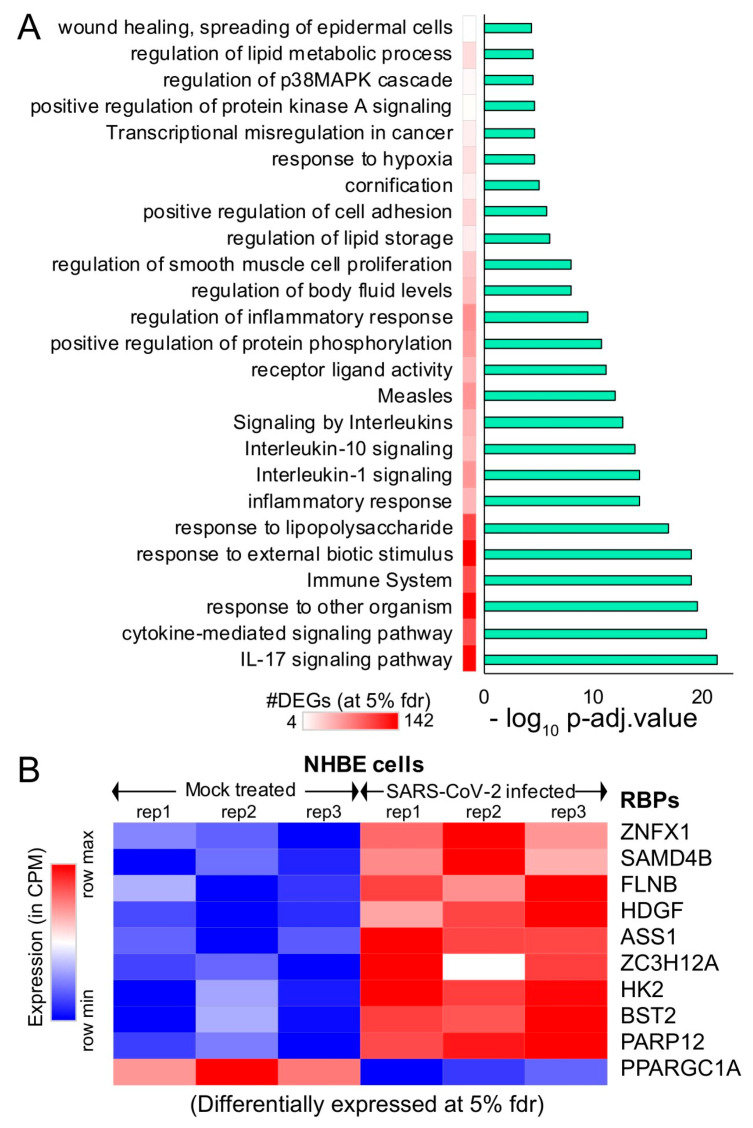
Differential expression analysis of mock-treated vs. SARS-CoV-2-infected primary human lung epithelial cells. (**A**) Bar plot illustrating the significant pathways obtained from the Gene Ontology (GO) term-based functional grouping of Differentially Expressed Genes (DEGs) at 5% False Discovery Rate (FDR) using ClueGO analysis (Cytoscape plugin) (**B**) Row normalized expression profile of differentially expressed RBPs in mock-treated and SARS-CoV-2-infected primary human lung epithelial cells (in biological triplicates). NHBE: normal vs. SARS-CoV-2-infected human bronchial epithelial cells.

Due to the rapidly evolving nature of SARS-CoV-2, the transcriptomic alterations contributed by the virus in humans remain unclear. To gain insight into the effect of SARS-CoV-2 infection on the host gene expression, we obtained the raw RNA sequencing data in normal vs. SARS-CoV-2-infected human bronchial epithelial (NHBE) cells [64] deposited in Gene Expression Omnibus (GEO) [65]. We investigated the gene expression levels between the mock-treated vs. SARS-CoV-2-infected NHBE cells and identified 327 differentially expressed genes at 5% FDR (among which, ~67% showed >1.25-fold elevated expressions in SARS-CoV-2-infected NHBE cells, as shown in Supplementary Figure S1). We conducted functional pathways associated with these differentially expressed genes (Figure 2A and Supplementary Figure S1) and identified 327 differentially expressed genes (at 5% FDR) using ClueGO [66] and revealed an enrichment for the immune response, cytokine-mediated signaling, an inflammatory response, and metabolism-associated genes (Figure 2A and Supplementary Table S1).

We observed a significant enrichment for the interleukin (IL)-17 pathway-associated genes during SARS-CoV-2 infection (Figure 2A). Our observation of the overrepresented IL-17 pathway was in accordance with recent studies that show the overactivation of IL-17-producing Th17 cells during severe immune injury in SARS-CoV-2 patients [67,68,69]. In addition to this, a recent review summarized that targeting IL-17 is immunologically plausible as a therapeutic strategy to prevent acute respiratory distress syndrome (ARDS) during SARS-CoV-2 infection, based on previous evidence that mice lacking functional IL-17 receptor *(Il17ra−/−)* signaling were shown to be more susceptible than wild-type mice to secondary pneumonia following infection with influenza A [70,71]. Thus, our analysis demonstrates the key role of several pathways, including IL-17 cytokine, response in SARS-CoV-2-infected cells.

An additional analysis also provided parallel support for the dysregulation of multiple RNA-binding proteins (FLNB, HDGF, ASS1, ZC3H12A, HK2, BST2, and PPARGC1A) involved in the immune response, cytokine-mediated signaling, and metabolism, when RNA-sequencing data from mock-treated vs. SARS-CoV-2-infected lung cells was employed (Figure 2B). The dysregulated expression of multiple RBPs implicated in vital cellular functions suggests that the virus may hijack the host cellular machinery by modulating the expression of key RBPs. We also observed that six of these differentially expressed genes that encode for RBPs were involved in at least 30% of the overrepresented pathways. Overall, our results imply that differentially expressed RNA-binding proteins in SARS-CoV-2-infected cells may contribute to alterations in the post-transcriptional regulatory networks governed by them.

### 2.3. Alternative Splicing Analysis Revealed an Abundance of Skipped and Mutually Exclusive Exons in Human Transcripts during SARS-CoV-2 Infection in Lung Epithelial Cells

Alternative splicing is a principal mechanism that contributes to protein diversity in eukaryotes, while regulating physiologically important immune responses during bacterial and viral infections [72]. Viral infections have been shown to cause global changes in the alternative splicing signatures in infected cells that may arise due to intrinsic factors like polymorphism at the splice sites or due to direct intervention by virulence factors [73,74,75]. A previous study on virus-host interactions has demonstrated that the human coronavirus targets various signaling pathways of ER stress, resulting in differential splicing outcomes [76]. Another study has shown that deletion of the E protein in recombinant SARS-CoV resulted in significant XBP1 gene splicing and a higher rate of apoptosis, suggesting that coronavirus-infected cells are susceptible to differential splicing events [77]. Therefore, we next investigated the alternative splicing events in mock vs. SARS-CoV-2-treated NHBE cells using rMATS (replicate Multivariate Analysis of Transcript Splicing) [78] (see Section 3).

**Figure 3:**
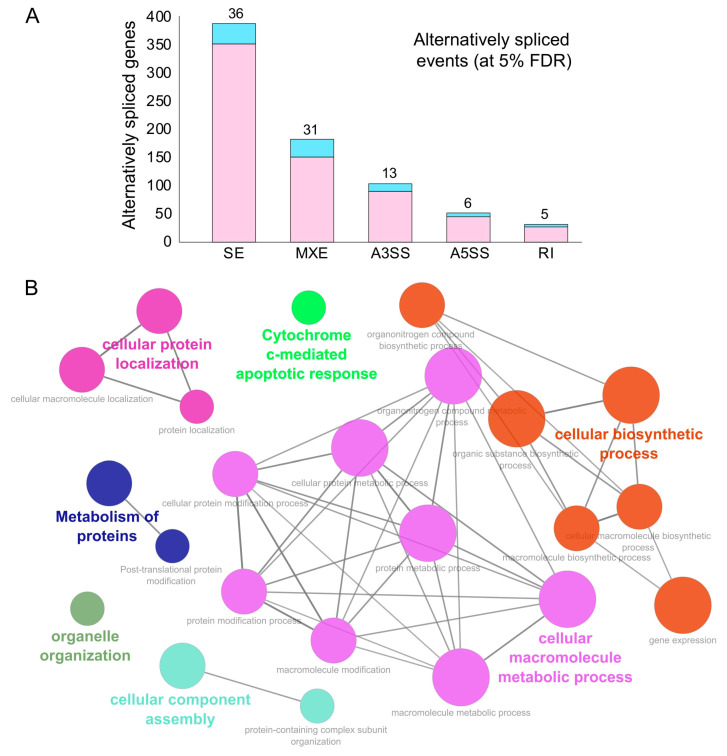
Alternative splicing events during SARS-CoV-2 infection. (**A**) Bar plot showing the genes (RBP-encoding genes in blue) exhibiting alternative splicing during SARS-CoV-2 infection in primary human lung epithelial cells (at 5% FDR). (**B**) Clustered GO term network obtained from the function annotation analysis and grouping of the GO term for the genes exhibiting alternative splicing using ClueGO (Cytoscape plugin). Significant clustering (adj. *p* < 1 × 10^−5^) of functional groups were color-coded by functional annotation of the enriched GO biological processes, with the size of the nodes indicating the level of significant association of genes per GO term were shown.

Our analysis revealed an abundance for skipped and mutually exclusive exonic events in the genes exhibiting alternative splicing events during SARS-CoV-2 infection at 5% FDR (Figure 3A and Supplementary Table S2). We also observed that 81 of the alternatively spliced genes encoded for RBPs (indicated in blue, Figure 3A) and, hence, could result in altering the downstream post-transcriptional regulatory networks in SARS-CoV-2-infected cells (Figure 3A).

These findings enhanced our mechanistic understanding of SARS-CoV-2-induced alternative splicing dysregulation in human cells and could be critical for developing novel therapeutic strategies. Next, we identified the functional annotation of the enriched GO terms related to the genes exhibiting alternative splicing using ClueGO [66]. Our data revealed that the majority of these genes were enriched for vital biological processes, including cellular protein localization, protein metabolism, organelle organization, cellular biosynthetic process, cellular component assembly, and cytochrome c-mediated apoptotic response (Figure 3B and Supplementary Table S3). These findings suggest that processes that contribute to the cell structure, interaction, and growth upon SARS-CoV-2 infection via alternatively spliced genes could contribute to rewiring of the post-transcriptional network. In summary, this analysis provided a clustered network of enriched biological functions that could be significantly dysregulated in SARS-CoV-2-infected cells.

### 2.4. Motif Enrichment Analysis Reveals Potential Human RBPs Titrated by the SARS-CoV-2 Viral Genome

**Figure 4:**
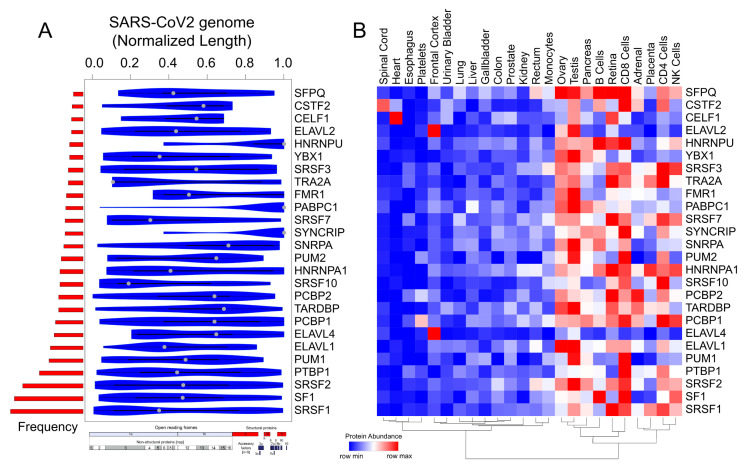
Motif enrichment analysis reveals potential human RBPs titrated by the SARS-CoV-2 viral genome. (**A**) Violin plot shows the statistically significant (*p* < 1 × 10^−5^) preferential binding profile of the RBP motifs (sorted by frequency of binding and greater than 10 sites) across the SARS-CoV-2 viral genome (length normalized) identified using FIMO. (**B**) Hierarchically clustered heatmap showing the protein abundance (row normalized) of RBPs across tissues.

The SARS-CoV-2 genome is the largest among the coronavirus family (~30kb) and is attributed to enhanced virus pathogenicity in the newly evolved strains of the COVID-19 pandemic [18,79]. Among the host-derived cellular factors, RBPs have been recognized as active participants in all steps of a viral infection [34,80,81]. A recent review has shown the linkage of 472 human proteins with viruses through unconventional RNA-binding domains [81]. However, the role of RNA-binding proteins during viral pathogenesis has remained an unappreciated domain in viral research. Therefore, in the present research, we conducted a systematic and comprehensive bioinformatic study to investigate the RBPs that could potentially bind on the RNA genome of SARS-CoV-2 by the motif enrichment analysis of human RBPs using Find Individual Motif Occurrences (FIMO) tool [82] (see Section 3). The motif analysis for RBPs with established position-specific weight matrices (PWMs) revealed a significant number of binding sites spread across the SARS-CoV-2 genome, illustrating the possible titration of post-transcriptional regulators by viral genome (Figure 4A, Supplementary Figure S2, and Supplementary Table S4). To date, the PWMs are available only for a small fraction of the experimentally known human RBPs [83]. Thus, our analysis represents an understanding of the post-transcriptional interactions for ~7.5% of the total RBPs collected from multiple studies [24,55,84]. Importantly, the binding pattern of the RBP motifs across the entire normalized length of the virus suggests that several human RBPs could be titrated across the viral genome (Figure 4A and Supplementary Table S4). Our results showed an enrichment for RBPs such as SRSFs, PCBPs, ELAVs, and HNRNPs being most likely to get sponged on the viral genome (Figure 4B). Our observation that specific RBPs such as SRSF7, HNRNPA1, and TRA2A with a well-known role in splicing exhibiting binding sites on SARS-CoV-2 RNA is in agreement with a recently published study [85]. We found that most of these RBPs were abundantly expressed in gonadal tissues; adrenal tissues; the pancreas; and immune cells, including B cells, CD4+ T cells, and NK cells (Figure 4B). Our analysis revealed that several members of the SRSF family (SRSF 1, 2, 3, 7, and 10) were potentially sponged by the SARS-CoV-2 genome. Although SRSF2 has been reported to be predominantly nuclear, there is evidence for other SRSFs being present in the cytoplasmic compartment by nuclear to cytoplasmic shuttling [86,87,88,89,90,91]. Therefore, such a cytoplasmic binding and sequestration of SRSFs by SARS-CoV-2 RNA could contribute to the dysregulation of host RNA targets. Furthermore, previous reports indicate that plus-stranded RNA viruses, such as polioviruses, utilize cellular factor PCBP for its translation and replication [44]. Therefore, we speculate that SARS-CoV-2 could also utilize host cell RBPs such as PCBPs to facilitate its genomic replication and translation. In addition to splicing factors and poly (rC)-binding proteins, other cellular RBPs such as ELAV1 that regulates the stability of the host transcripts could also stabilize viral RNAs by sequestration on the viral genome [81,92]. In summary, these findings suggest that SARS-CoV-2 could sponge human RBPs on its RNA, resulting in an altered post-transcriptional gene regulatory network in the host cells. Targeting host proteins has been appreciated as an effective strategy to combat a wide range of viral infections, and therefore, an understanding of the potential RBPs that are likely sponged by the viral genome is crucial to develop novel therapeutics [35].

### 2.5. SARS-CoV-2 Genome Titrates the Abundance of Functionally Important miRs in Human Tissue

**Figure 5:**
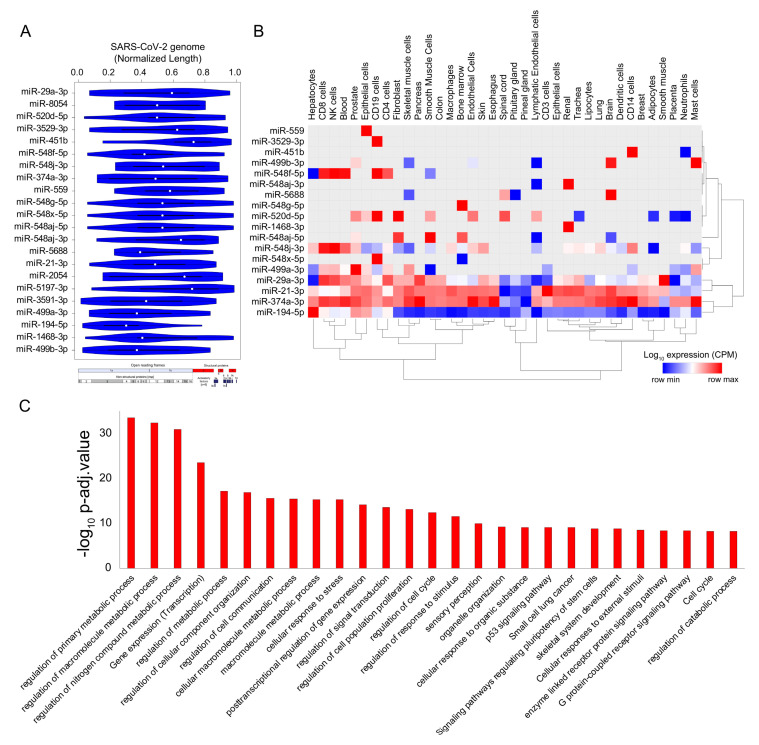
SARS-CoV-2 genome titrates the abundance of functionally important micro-RNAs (miRs) in human tissue. (**A**) Violin plot shows the statistically significant (*p* < 1 × 10^−5^) preferential binding profile of miR motifs (sorted by frequency of binding >15 sites) across the SARS-CoV-2 viral genome (length normalized) identified using FIMO. (**B**) Hierarchically clustered heatmap showing the log10 expression (Copies Per Million mapped reads (CPM), row normalized) of miRs across the tissues. (**C**) Bar plot illustrating the significant biological processes obtained from the gene ontology enrichment-based functional grouping of miR target genes (obtained from miRNet). Significant clustering (adj. *p* < 1 × 10^−10^) of genes enriched in GO biological processes generated by ClueGO analysis (Cytoscape plugin).

Micro-RNAs (miRs) are small noncoding RNA molecules that function as central regulators of post-transcriptional gene regulation. Human miRs have been associated with a variety of pathophysiological pathways and demonstrate differential expression during viral infections [93,94]. Recently, a few computational studies have shed light on the interplay between human miRs and SARS-CoV-2 target genes, indicating a crucial role in regulating the viral load in host cells [95,96]. However, a comprehensive understanding of the functional role of host miRs during SARS-CoV-2 infection has remained elusive until now. Recently, a machine learning-based study predicted that miRs could impact SARS-CoV-2 infection through several mechanisms, such as interfering with replication, translation, and by modulation of the host gene expression [95]. In this study, we used a computational approach to investigate the potential binding sites of human miRs in the SARS-CoV-2 genome using FIMO [82]. We identified 22 miRNAs that could potentially bind throughout the length of the SARS-CoV-2 viral genome (Figure 5A and Supplementary Table S5). Among the human miRs likely to be sequestered on the SARS-CoV-2 genome, miR-374a-3p has recently been predicted to target the SARS-CoV-2 gene; in particular, it has been shown to target the gene encoding the spike protein, which is essential for virus entry into the host cell [95,97]. In addition to targeting the spike protein-encoding genes, miR-374a-3p is also predicted to target the ORF1ab in the SARS-CoV-2 genome that encodes for the 5′viral replicase, based on the function similarity of SARS-CoV-2 coding genes with SARS-CoV [98]. Our data suggests that miRs can be bound by SARS-CoV-2 RNA, supporting a likely model where host miRs can be sponged by SARS-CoV-2 and thereby contributing to the decreased binding of miRs to the human mRNA targets, resulting in altering the expression patterns of human genes. Although less likely, it is also possible that nuclear preprocessed miRNAs complimentary to SARS-CoV-2 RNA could be sequestered by viral RNA, and because of this binding, miRNAs may not be able to do post-transcriptional gene silencing, thereby increasing the target gene expression. In either case, these results suggest that several important miRs are likely being titrated by the SARS-CoV-2 genome that could result in the dysregulation of post-transcriptional networks in the infected cells and could be attributed to viral pathogenesis. However, these results require experimental validation to conclude their role in SARS-CoV-2 infection.

The majority of the identified miRs were highly expressed in immune cells, including CD8+T cells, CD4+T, NK cells, CD14 cells, and mast cells, suggesting that these miRs might contribute to alter post-transcriptional regulation in specialized immune cells and assist in the progression of the viral infection and host immune response across other human tissues (Figure 5B). Our results also indicate that the highly confident genes targeted by these sponged miRs were enriched for functional themes, including the “regulation of metabolic processes”, “post-transcriptional gene regulation”, and “cell-to-cell communication” suggestive of a large-scale dysregulation across tissues (Figure 5C and Supplementary Table S6). Overall, our results present a comprehensive analysis of the miRs being potentially titrated on the viral genome, resulting in altered post-transcriptional gene regulation. These findings provided enhanced our understanding of the miR-associated mechanism in SARS-CoV-2 pathogenesis and could provide important clues for designing RNA-based therapeutics.

## 3. Materials and Methods

### 3.1. Dissection of SARS-CoV-2 Proteins Interacting with Human RBPs

We obtained the high confidence mass spectrometry-based SARS-CoV-2 viral protein to human protein interaction network established by Gordan et al., 2020 [23] in HEK293 cells. We dissected the human RBPs directly interacting with viral proteins and integrated with first-neighbor-interacting RBPs (obtained from BioGRID [56]). We also extracted the protein abundance of these SARS-CoV-2-interacting RBPs across human tissues from the human proteome map [57]. The abundance of these proteins were hierarchically clustered and row normalized and represented as a heatmap generated from Morpheus (https://software.broadinstitute.org/morpheus/).

### 3.2. Differential Expression Analysis of Mock-Treated vs. SARS-CoV-2-Infected Primary Human Lung Epithelial Cells

We downloaded the raw RNA sequencing data deposited in Gene Expression Omnibus (GEO) [65]. Specifically, we downloaded the paired-end raw sequencing (FASTQ) files of mock-treated and SARS-CoV-2 (USA-WA1/2020)-infected primary human lung epithelial cells (in biological triplicates) using the Sequence Read Archive (SRA) Toolkit (fastq-dump command) from the GEO cohort GSE147507 [64]. The quality of the sequence reads was ensured using the FASTX-Toolkit (http://hannonlab.cshl.edu/fastx_toolkit/), with a minimum of Phred quality score 20 for each sample. We processed the raw sequencing reads using the in-house NGS data processing pipeline, as described previously [99,100]. Briefly, we used Hierarchical Indexing for the Spliced Alignment of Transcripts (HISAT) [101] for aligning the short reads from the RNA-seq experiments onto the human reference genome (hg38). SAM (Sequence Alignment/Map) files obtained from HISAT were postprocessed using SAMtools (version 0.1.19) [102,103] for converting SAM to BAM (Binary Alignment/Map), followed by sorting and indexing the output BAMs. The sorted BAM files were parsed using the python script provided by StringTie (version 1.2.1) [104] to obtain the count matrix for gene levels across the samples. This count matrix was used to perform the differential expression analysis between mock vs. SARS-CoV-2-infected NHBE cells using DE-seq2 [105]. Statistically significant (at 5% FDR) differentially expressed genes were collected for downstream data analysis. Functional enrichment analysis of these genes was performed with a *p*-value threshold < 10^−10^ using ClueGO [66] (a Cytoscape [106] plugin) and represented as a bar plot illustrating the significant pathways obtained from the GO term-based functional grouping of differentially expressed genes.

### 3.3. Identification of Alternative Splicing Events during SARS-CoV-2 Infection

We used rMATS (replicate Multivariate Analysis of Transcript Splicing) [78] to identify differential alternative splicing (AS) events between the mock vs. SARS-CoV-2-treated NHBE cells. rMATS used sorted BAM (Binary Alignment/Map) files, obtained from aligning the fastq files against the hg38 reference genome using HISAT (as discussed above). It also uses a GTF file (gene transfer file format), downloaded from Ensembl (version 97) [107] for the existing annotation of exons. Briefly, rMATS enabled the analysis of the inclusion/exclusion of target exons/introns, contributing to different types of alternative splicing events, namely skipped exon (SE), alternative 5′ splice site (A5SS), alternative 3′ splice site (A3SS), mutually exclusive exons (MXE), and retained intron (RI), between pairs of conditions and provided the difference in the level of inclusion of an exon denoted by the Percentage Splicing Index (ψ score) (as described previously [99]). Genes exhibiting alternatively spliced events detected below the 5% FDR threshold were documented in Supplementary Table S2. Functional enrichment analysis of these genes was performed using ClueGO [66] and documented in Supplementary Table S3.

### 3.4. Identification of Potential Binding Blocks of RBPs in the SARS-CoV-2 Viral Genome Using Motif Enrichment Analysis

We obtained the RBP motifs from ATtRACT [83] and scanned across the SARS-CoV-2 viral genome using FIMO [82,108], with default parameters. Resulting genomic locations for each RBP motif were documented in Supplementary Table S4. For each binding motif, the scanned genomic location was normalized by considering the mid-point of the genomic coordinate divided with the SARS-CoV-2 genome length. Additionally, the occurrence of each RBP motif binding across the viral genome was computed. Statistically significant (*p*-value < 1 × 10^−5^) preferential binding profile of the RBP motifs (sorted by frequency of binding and greater than 10 sites) across the SARS-CoV-2 viral genome (length normalized) identified using FIMO [82] was visualized in the violin plot. Additionally, the protein abundance of the corresponding RBPs were extracted from the human proteome map [57] and represented as hierarchically clustered heatmaps across the tissues.

### 3.5. Identification of the Potential Binding Blocks of micro-RNAs in the SARS-CoV-2 Viral Genome Using Motif Enrichment Analysis

We obtained the miR motifs from MEME-suite [108] and scanned across the SARS-CoV-2 viral genome using FIMO [82,108], with default parameters. Resulting genomic locations for each miR motif are documented in Supplementary Table S6. For each miR motif, the scanned genomic location was normalized by considering the mid-point of the genomic coordinate divided with the SARS-CoV-2 genome length. Additionally, the occurrence of each miR motif binding across the viral genome was computed. Statistically significant (*p* < 1 × 10^−5^) preferential binding profile of miR motifs (sorted by frequency of binding and greater than 15 sites) across the SARS-CoV-2 viral genome (length normalized) identified using FIMO was visualized in a violin plot. Additionally, the expression profile of the corresponding miRs were extracted from the FANTOM5 project [109] and represented as hierarchically clustered heatmaps across the tissues. To understand the generic biological function of these miRs that could be altered by being titrated by the SARS-CoV-2 genome in the host cells, we downloaded the high-confidence miR target genes (obtained from miRNet [110,111]) and performed a function annotation analysis. Resulting significant biological processes, obtained from the gene ontology enrichment-based functional grouping of these miR target genes, were illustrated in a bar plot. Significant clustering (adj. *p* < 1 × 10^−10^) of genes enriched in GO biological processes was generated by ClueGO [66] analysis (a Cytoscape [106] plugin).

## 4. Conclusions

Our analysis integrates a comprehensive interaction network to map the immediate interactions between the SARS-CoV-2 genome and proteome with human post-transcriptional regulators such as RBPs and miRNAs, along with their tissue-specific expressions and functional annotations. Given the importance of developing effective therapeutic strategies in the current pandemic, understanding the effect of SARS-CoV-2 infection on the human transcriptional and post-transcriptional regulatory networks is crucial for identifying effective drug targets. To delineate the impact of SARS-CoV-2 on the host cells’ post-transcriptional gene regulatory network, we integrated the interactions between SARS-CoV-2-encoded proteins with human RNA-binding proteins. Our findings indicated 51 human RBPs (including PABP-4, DDX21, DDX10, and EIF4H) that interact directly with the viral structural and nonstructural proteins that, in turn, interact indirectly with ~65% other secondary neighbor RBPs. Thus, these findings suggest a comprehensive network of human RBPs and SARS-CoV-2 proteins that could alter the post-transcriptional regulatory mechanisms at several layers in the infected cells.

We showed that the expression profiles of the majority of the directly interacting RBPs were associated with gonadal tissues and immune cell types. Our study also highlighted that several of the differentially expressed genes in SARS-CoV-2-infected cells were enriched for biological pathways, such as the immune response, cytokine-mediated signaling, the inflammatory response, and metabolism-associated genes that are indispensable for cell survival. Importantly, we found that, among the differentially expressed genes, six RBP-encoding genes contribute to the functionally important pathways in the host cells, implying a potential impact of SARS-CoV-2 infection on post-transcriptional regulation. Further, our analysis demonstrated the abundance of skipped exonic and mutually exclusive exonic events in SARS-CoV-2-infected cells, suggesting these alternative splicing events as a plausible cause for the altered post-transcriptional regulation in human cells. Using the motif enrichment analysis, we show that two key classes of post-transcriptional regulators, RBPs and miRs, are likely to be titrated by the SARS-CoV-2 genome, which could result in the systemic dysregulation of post-transcriptional networks in the infected cells. Currently, there are no effective antiviral therapies available for COVID-19. Therefore, our analyses provide a roadmap to enhance the understanding of the potential interactions of SARS-CoV-2 with key post-transcriptional regulators in the human genome.

## Supplementary Materials

Supplementary materials can be found at https://www.mdpi.com/1422-0067/21/19/7090/s1.

## Abbreviations

SRSF	Serine and arginine Rich Splicing Factors
PCBP	Poly(rC)-Binding Protein
ELAV	Embryonic Lethal Abnormal Visual system
HNRNP	Heterogeneous Nuclear Ribonucleoproteins
GO	Gene Ontology
FDR	False Discovery Rate
MEME	Multiple Em for Motif Elicitation
FIMO	Find Individual Motif Occurrences
NGS	Next Generation Sequencing
