# Supplementary material for: Role of SARS-CoV-2 in Altering the RNA-Binding Protein and miRNA-Directed Post-Transcriptional Regulatory Networks in Humans
Source: Int J Mol Sci. 2020 Sep 25;21(19):7090. doi: 10.3390/ijms21197090 (PMC7582926; doi:10.3390/ijms21197090)
Supplement: Supplementary file 1 [file ijms-21-07090-s001.zip › Supps final/SARS-CoV-2_Supplementary_Materials.docx]

**Role of SARS-CoV-2 in altering the RNA binding protein and miRNA directed post-transcriptional regulatory networks in humans**

Rajneesh Srivastava^1^, Swapna Vidhur Daulatabad^1^, Mansi Srivastava^1^*, Sarath Chandra Janga^1,2,3^*

^1^Department of Biohealth Informatics, School of Informatics and Computing, Indiana University Purdue University, 719 Indiana Ave Ste 319, Walker Plaza Building, Indianapolis, Indiana 46202

^2^Center for Computational Biology and Bioinformatics, Indiana University School of Medicine, 5021 Health Information and Translational Sciences (HITS), 410 West 10th Street, Indianapolis, Indiana, 46202

^3^Department of Medical and Molecular Genetics, Indiana University School of Medicine, Medical Research and Library Building, 975 West Walnut Street, Indianapolis, Indiana, 46202

** Correspondence can be addressed to :*

*Mansi Srivastava (Email: mansriva@iupui.edu)*

*or*

*Sarath Chandra Janga (Email: scjanga@iupui.edu)*

*School of Informatics and Computing*

*Indiana University Purdue University*

*719 Indiana Ave Ste 319*

*Indianapolis, Indiana 46202*

**SUPPLEMENTARY MATERIALS**

**Supplementary Figure Legends:**

**Figure S1.** Protein-protein interaction network of differentially expressed genes in mock treated versus SARS-CoV-2 infected primary human lung epithelium (NHBE cells) where node size and color show the absolute and relative log2 fold changes, respectively.

**Figure S2.** (A) Violin plot shows the statistically significant (p-value < 1e-05) preferential binding profile of RBP motifs (sorted by frequency of binding) across the SARS-CoV-2 viral genome (length normalized), identified using FIMO (B) Hierarchical clustered heatmap showing the protein abundance (row normalized) of RBPs across tissues.

**Supplementary Table Legends:**

**Table S1.** Significant biological pathways obtained from functional annotation analysis of differentially expressed genes (at 5% fdr) in mock treated versus SARS-CoV-2 infected primary human lung epithelium (NHBE cells) using ClueGO (a cytoscape plugin).

**Table S2.** Identification of alternative splicing events using rMATS (replicate Multivariate Analysis of Transcript Splicing) in mock treated versus SARS-CoV-2 infected primary human lung epithelium (NHBE cells).

**Table S2.** Significant biological pathways obtained from functional annotation analysis of alternatively spliced genes (at 5% fdr) in mock treated versus SARS-CoV-2 infected primary human lung epithelium (NHBE cells) using ClueGO (a cytoscape plugin).

**Table S4.** Preferential binding location of RBP motifs identified by FIMO across the SARS-CoV-2 viral genome.

**Table S5.** Preferential binding location of miR-motifs identified by FIMO across the SARS-CoV-2 viral genome.

**Table S6.** Significant biological pathways obtained from functional annotation analysis of mir-targeted genes (from mirNet) using ClueGO (a cytoscape plugin).
