# Supplementary figures and images for: Role of SARS-CoV-2 in Altering the RNA-Binding Protein and miRNA-Directed Post-Transcriptional Regulatory Networks in Humans
Source: Int J Mol Sci. 2020 Sep 25;21(19):7090. doi: 10.3390/ijms21197090 (PMC7582926; doi:10.3390/ijms21197090)

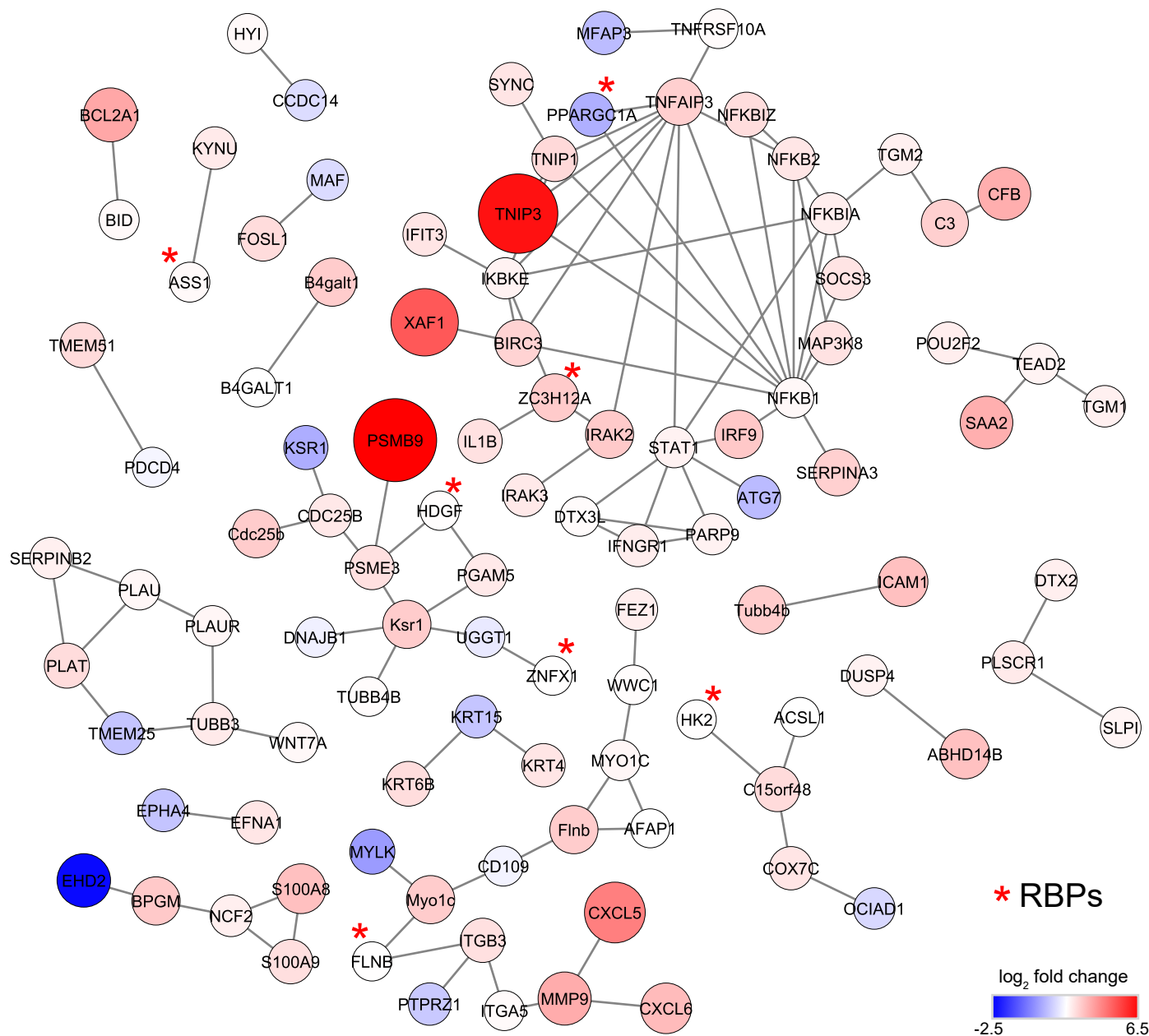

Supplement: Supplementary file 1 [file ijms-21-07090-s001.zip › Supps final/FigS1.pdf]
