# Supplementary material for: Role of SARS-CoV-2 in Altering the RNA-Binding Protein and miRNA-Directed Post-Transcriptional Regulatory Networks in Humans
Source: Int J Mol Sci. 2020 Sep 25;21(19):7090. doi: 10.3390/ijms21197090 (PMC7582926; doi:10.3390/ijms21197090)
Supplement: Supplementary file 1 [file FigS2.pdf › Supps final/FigS2.pdf]

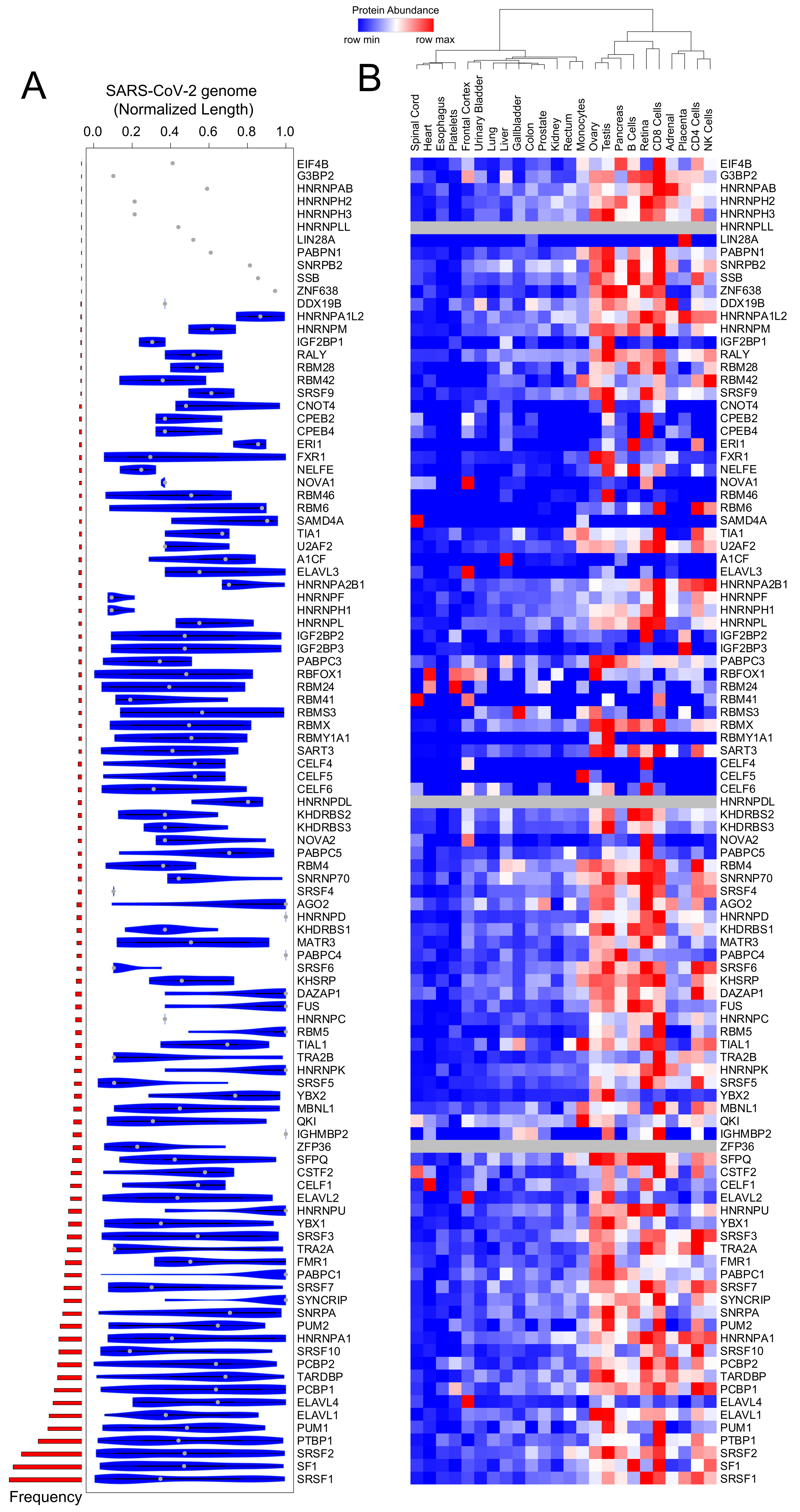

A
B
SARS-CoV-2 genome
(Normalized Length)
0.0 0.2 0.4 0.6 0.8 1.0
Frequency
Protein Abundance
row min
row max
Spinal Cord
Heart
Esophagus
Platelets
Frontal Cortex
Urinary Bladder
Lung
Liver
Gallbladder
Colon
Prostate
Kidney
Rectum
Monocytes
Ovary
Testis
Pancreas
B Cells
Retina
CD8 Cells
Adrenal
Placenta
CD4 Cells
NK Cells
EIF4B
G3BP2
HNRNPAB
HNRNPH2
HNRNPH3
HNRNPLL
LIN28A
PABPN1
SNRPB2
SSB
ZNF638
DDX19B
HNRNPA1L2
HNRNPM
IGF2BP1
RALY
RBM28
RBM42
SRSF9
CNOT4
CPEB2
CPEB4
ERI1
FXR1
NELFE
NOVA1
RBM46
RBM6
SAMD4A
TIA1
U2AF2
A1CF
ELAVL3
HNRNPA2B1
HNRNPF
HNRNPH1
HNRNPL
IGF2BP2
IGF2BP3
PABPC3
RBFOX1
RBM24
RBM41
RBMS3
RBMX
RBMY1A1
SART3
CELF4
CELF5
CELF6
HNRNPDL
KHDRBS2
KHDRBS3
NOVA2
PABPC5
RBM4
SNRNP70
SRSF4
AGO2
HNRNPD
KHDRBS1
MATR3
PABPC4
SRSF6
KHSRP
DAZAP1
FUS
HNRNPC
RBM5
TIAL1
TRA2B
HNRNPK
SRSF5
YBX2
MBNL1
QKI
IGHMBP2
ZFP36
SFPQ
CSTF2
CELF1
ELAVL2
HNRNPU
YBX1
SRSF3
TRA2A
FMR1
PABPC1
SRSF7
SYNCRIP
SNRPA
PUM2
HNRNPA1
SRSF10
PCBP2
TARDBP
PCBP1
ELAVL4
ELAVL1
PUM1
PTBP1
SRSF2
SF1
SRSF1
